# Enhancing motion tracking accuracy of a low-cost 3D video sensor using a biomechanical model, sensor fusion, and deep learning

**DOI:** 10.3389/fresc.2022.956381

**Published:** 2022-08-16

**Authors:** Shahar Agami, Raziel Riemer, Sigal Berman

**Affiliations:** Department of Industrial Engineering and Management, Ben-Gurion University of the Negev, Beer-Sheva, Israel

**Keywords:** kinematics, recurrent neural network (RNN), rehabilitation, upper limb, virtual reality

## Abstract

Low-cost 3D video sensors equipped with routines for extracting skeleton data facilitate the widespread use of virtual reality (VR) for rehabilitation. However, the accuracy of the extracted skeleton data is often limited. Accuracy can be improved using a motion tracker, e.g., using a recurrent neural network (RNN). Yet, training an RNN requires a considerable amount of relevant and accurate training data. Training databases can be obtained using gold-standard motion tracking sensors. This limits the use of the RNN trackers in environments and tasks that lack accessibility to gold-standard sensors. Digital goniometers are typically cheaper, more portable, and simpler to use than gold-standard motion tracking sensors. The current work suggests a method for generating accurate skeleton data suitable for training an RNN motion tracker based on the offline fusion of a Kinect 3D video sensor and an electronic goniometer. The fusion applies nonlinear constraint optimization, where the constraints are based on an advanced shoulder-centered kinematic model of the arm. The model builds on the representation of the arm as a triangle (the arm triangle). The shoulder-centered representation of the arm triangle motion simplifies constraint representation and consequently the optimization problem. To test the performance of the offline fusion and the RNN trained using the optimized data, arm motion of eight participants was recorded using a Kinect sensor, an electronic goniometer, and, for comparison, a passive-marker-based motion tracker. The data generated by fusing the Kinect and goniometer recordings were used for training two long short-term memory (LSTM) RNNs. The input to one RNN included both the Kinect and the goniometer data, and the input to the second RNN included only Kinect data. The performance of the networks was compared to the performance of a tracker based on a Kalman filter and to the raw Kinect measurements. The accuracy of the fused data was high, and it considerably improved data accuracy. The accuracy for both trackers was high, and both were more accurate than the Kalman filter tracker and the raw Kinect measurements. The developed methods are suitable for integration with immersive VR rehabilitation systems in the clinic and the home environments.

## Introduction

The availability of low-cost 3D video sensors suitable for joint motion tracking has paved the way for affordable motion analysis and motion rehabilitation training with virtual reality (VR) (1). Such low-cost, marker-less systems can be simply and rapidly installed in both clinic and home environments. However, the motion tracking accuracy of low-cost 3D video sensors is limited, especially for multiple joint motion tracking (2). The limited accuracy restricts the utility of the low-cost 3D video sensors for rehabilitation training and assessment, especially for upper limb rehabilitation. The increased difficulty of tracking upper limb motion stems from the complexity and the variability of upper limb motion profiles. Developing algorithms for improving the accuracy of upper limb motion tracking using low-cost 3D video sensors is thus a crucial yet challenging task.

The Kinect (Microsoft, USA) sensor is among the most used low-cost 3D video sensor suitable for full-body motion tracking (1, 3). One reason for the Kinect's high popularity is that a custom, full-body skeleton extraction method is part of the Kinect's software development kit. The accuracy of the extracted joint positions and angles using the software has been extensively studied with various methods and in different scenarios (3–7). The accuracy was found to vary and depend on the camera location (orientation angle and distance), the use case (e.g., the existence of obstacles, the direction of movement), and the joint tracked. For example, for a walking task away from and toward the camera, the correlation of joint angles measured using a Kinect sensor (both Kinect 1 and Kinect 2) and those measured with the gold-standard sensor (Vicon system) was found to be moderate to poor for all joints, except for the knees (for which there was good to excellent correlation) (7).

Several tracking algorithms have been suggested for improving the motion tracking accuracy of low-cost 3D video sensors. The commonly used tacking algorithms include the classical Kalman filter (8, 9) and more recently neural networks (10). Recurrent neural networks (RNNs) are especially suitable network structures for processing time-series data, e.g., joint motion data, and have been used for tracking upper limb motion based on Kinect data (11–14). Excellent accuracy improvement can be obtained with neural networks, especially RNNs, in environments and tasks for which the network was trained. Training datasets are produced by recording motion using gold-standard motion tracking systems parallel to the low-cost 3D video. Accuracy improvement across environments and tasks requires large training datasets which are not currently available; therefore, the trained RNNs are specific to the environment and the use case for which their training data was acquired. This requirement for large training sets produced with gold-standard systems constitutes a stumbling block to the large-scale implementation of advanced VR systems based on low-cost 3D video sensors in clinics and home environments.

The accuracy of motion data from low-cost 3D sensors can be improved using offline motion data optimization methods. The methods suggested typically apply noise filtering methods and integrate biomechanical constraints (2). The biomechanical constraints can be specific or floating constraints, i.e., related or unrelated to an environment or a task. For example, to improve the estimation of lower limb and trunk kinematics, Matthew et al. (2) applied both floating biomechanical constraints based on body segment length and task-specific constraints fixing the ankle position in a sit-to-stand task. However, due to the variability of upper limb motion defining motion constraints suitable for optimizing joint position measurement accuracy is challenging. In the current research, we suggest an advanced upper-limb rigid body model based on a shoulder-centered representation of the motion of the arm as the rigid motion of a triangle (the arm triangle). Using a shoulder-centered frame for optimization enables using data collected from multiple locations of the Kinect camera with respect to the participant. Digital goniometers are cheaper, more portable, and simpler to use than gold-standard motion tracking sensors. Using the arm triangle model facilitates fusing data recorded from a Kinect and a digital goniometer based on task constraints suitable for common upper-limb rehabilitation training protocols for subjects with stroke.

Stroke is a leading cause of long-term sensory-motor disability (15, 16). Upper limb impairment following stroke is highly prevalent (17, 18), and many upper-limb motion rehabilitation training protocols that integrate VR have been developed for stroke survivors (12, 19–21). Facilitating the widespread use of low-cost 3D video sensors for motion rehabilitation for patients with stroke can assist in improving accessibility to rehabilitation treatment. Individuals with stroke tend to use motion compensations, e.g., excessive trunk motion during reaching motion. Therefore, many training protocols involve the restriction of trunk motion during rehabilitation training (22). Individuals with stroke tend to suffer from muscle weakness. Therefore, a passive or active mechanism is typically used to support the arm against gravity during training (23–27); for example, the forearm is strapped to a manipulandum supporting motion against gravity. In many cases, such support leads to immobilizing elbow supination pronation motion. Such a motion restriction is suitable for practicing reaching and pointing motion. The task-specific constraints applied in the current work assume an upper-limb rehabilitation system designed for practicing reaching and pointing motion with restrained trunk motion and with a manipulator supporting the forearm and hand against gravity, i.e., immobilizing elbow supination pronation motion. These task-specific constraints are integrated within the triangle-based rigid body model of the arm.

Fusing the Kinect and goniometer measurements using the offline optimization method developed in the current work facilitates the generation of highly accurate joint location estimates, which can be used as training data for training an RNN for upper limb motion tracking. Section two presents the developed methods, which include the shouldered-centered arm-triangle kinematic model, the constrained optimization developed for fusing the Kinect with a goniometer, and the RNN tracker. Section three represents an experiment conducted to validate the methods with motion recorded from eight healthy subjects. Section four presents the results, and Section five presents a discussion of the results. Finally, conclusions are presented in Section six.

## Methods

### Kinematic model of the arm

**Figure 1:**
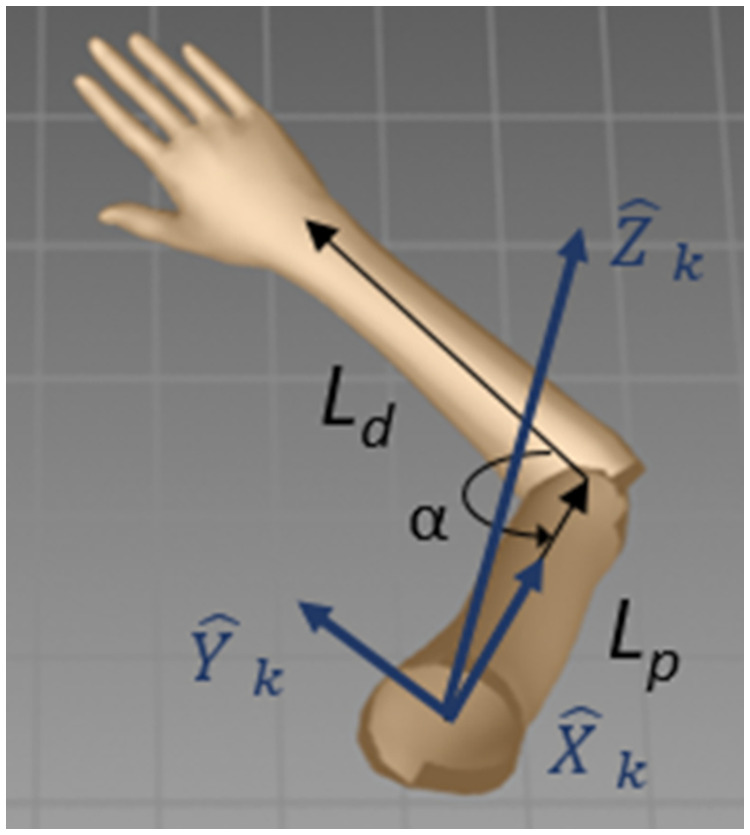
Shoulder coordinate frame. *L*_d_ is the distal link. *L*_p_ is the proximal link. $\;{\hat{x}}_{k}$,$\;{\hat{y}}_{k}$, and $\;{\hat{z}}_{k}$ are the shoulder coordinate frame axes. *α* is the elbow extension angle (the triangle angle).

The arm can be modeled as a system of two rigid links, a distal link, *L*_d_, connecting the wrist and the elbow and a proximal link, *L*_p_, connecting the elbow and the shoulder (Figure 1). The links are connected to two joints, a shoulder joint with three degrees of freedom (flexion–extension, abduction–abduction, internal–external rotation) and an elbow joint with two degrees of freedom (flexion–extension, supination–pronation). The two links of the arm and the vector connecting the shoulder and the wrist joints form the arm triangle (28, 29). The angle between the links, *α*, is the elbow extension angle, also termed the triangle angle. When elbow pronation–supination is immobilized, arm motion can be modeled as a change in the triangle angle together with a 3D rotation of the triangle about the shoulder.

**Figure 2:**
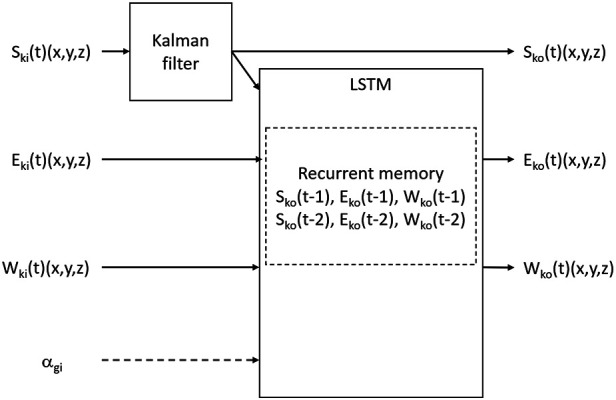
LSTM RNN with a Kalman filter for the shoulder joint location. The network receives measurements from the sensors and has a recurrent memory of two previous time steps. S: shoulder, E: elbow, W: wrist, *α*: elbow angle, k: Kinect frame, g: goniometer, i: input, o: output, t: time. One network included the goniometer measurement as input, and the other did not.


The arm triangle facilitates straightforward integration of the elbow extension angle, which can be measured by a goniometer, *α*_g_, and the joint locations extracted by the Kinect skeleton model from the 3D image. In the Kinect coordinate frame, the joint locations are the wrist, *W*_k_, elbow, *E*_k_, and shoulder, *S*_k_. These locations form the shoulder–elbow vector, *SE*_k _=_ _*E*_k_ − *S*_k_, and the elbow–wrist vector, *EW*_k _=_ _*W*_k_ − *E*_k_. A shoulder-centered coordinate frame, i.e., a coordinate frame with an origin at the shoulder (*S*_k_), is defined such that the $\;{\hat{x}}_{k}$-axis is along the direction of the *SE*_k_ vector, the $\;{\hat{z}}_{k}$-axis is orthogonal to the arm triangle plane (in the direction of $SE_{k}\times EW_{k}$), and the $\;{\hat{y}}_{k}$-axis is defined as their orthogonal complement forming a right-handed coordinate frame (Figure 2). In this shoulder coordinate frame, the elbow coordinates are *E*_s_*_ _*=*_ _*[*L*_p_, 0, 0], and the wrist coordinates are *W*_s_*_ _*=* *[*L*_p_ − *L*_d_cos(*α*), *L*_d_sin(*α*), 0]. A homogeneous shoulder-Kinect transformation matrix, *T*_ks_, transforms point coordinates from the shoulder coordinate frame to the Kinect coordinate frame:

(1) $$T_{\mathrm{ks}}=\left[\begin{array}{cc} {\hat{x}}_{k} & \begin{array}{ccc} {\hat{y}}_{k} & {\hat{z}}_{k} & S_{k} \end{array}\\ 0 & \begin{array}{ccc} 0 & 0 & 1 \end{array} \end{array}\right]$$

### Optimization

The shoulder joint position is determined based on the Kinect measurement. Since trunk motion is restricted, the shoulder location is expected to remain constant. To allow for some shoulder motion, the location of the shoulder joint is determined using a Kalman filter, modeling no shoulder motion (zero motion speed). The elbow and wrist joint positions are determined by optimally fusing the measurements of the Kinect and the goniometer. Since angle measurements from the goniometer are typically more accurate than the Kinect joint position measurements, the optimization gives preference to the goniometer. The arm triangle angle is set to the angle measured by the goniometer, and then, the optimal elbow and wrist locations are found by rotating the arm triangle about the shoulder while minimizing the error between the locations of the triangle apexes and the location measured by the Kinect sensor.

Constrained nonlinear optimization of multivariate scalar functions is applied for finding the rotation of the arm triangle about the shoulder that minimizes the sum of the Euclidean distances to the measured elbow and wrist locations. The optimization is conducted for each time step based on the shoulder, elbow, and wrist joint locations measured by the Kinect, the angle measured by the goniometer, and the shoulder, elbow, and wrist locations determined in the previous optimization time step. The joint location determined by the optimization is subsequently used for the next time step.

The axis–angle (30) representation is used for rotating the arm triangle during the optimization. Constraints are imposed on the rotation angle, *θ*, and the direction of the rotation axis, ${\hat{v}}_{s}$. The rotation angle is constrained to be small, and the rotation axis is constrained to be similar to the axis orthogonal to the arm triangle plane (the $\hat{z}\;$-axis of the shoulder coordinate frame). The rotation angle is constrained to be small based on the motion sampling frequency and the expected movement speed. The rotation axis is constrained to be similar to the $\hat{z}\;$-axis since the motion task is reaching or pointing. For reaching and pointing tasks, arm orientation is not prescribed by the task. Research has shown that for reaching and pointing movement, changes in arm orientation throughout the movement are very small (28). The optimization model can be solved using sequential quadratic programming (31, 32). At each iteration, a step rotation matrix, $R_{\mathrm{step}}\left(\theta ,\;{\hat{v}}_{s}\right)$, is calculated using the axis–angle representation. The distance vectors between the calculated and the measured joint locations, *e*_E_ (elbow error) and *e*_W_ (wrist error) at time step *t*, are

(2) $$\left[\begin{array}{c} e_{E}(t)\\ 1 \end{array}\right]=\left[\begin{array}{c} E_{k}(t)\\ 1 \end{array}\right]-T_{\mathrm{ks}}(t-1)\cdot \left[\begin{array}{c} R_{\mathrm{step}}\cdot E_{s}(t-1)\\ 1 \end{array}\right]$$

(3) $$\left[\begin{array}{c} e_{W}(t)\\ 1 \end{array}\right]=\left[\begin{array}{c} W_{k}(t)\\ 1 \end{array}\right]-T_{\mathrm{ks}}(t-1)\cdot \left[\begin{array}{c} R_{\mathrm{step}}\cdot W_{s}(t-1)\\ 1 \end{array}\right]$$

The Euclidean distances ${\bar{e}}_{E}$ and ${\bar{e}}_{W}$ are calculated from the distance vectors, and the sum of the squared distances is minimized. The optimization model is defined by

(4) $$\begin{array}{cc} \mathrm{minimize} & \;{\bar{e}}_{E}^{2}+{\bar{e}}_{W}^{2}\;\\ \mathrm{over} & \theta \in R,\;\;{\hat{v}}_{s}\in R^{3}\\ \begin{array}{c} \mathrm{subject}\;\mathrm{to} \end{array} & \begin{array}{c} \theta \leq 1^{\circ }\\ ||{\hat{v}}_{s}||=1\\ \mathrm{arcos}(\hat{z}\cdot {\hat{v}}_{s})<30^{\circ } \end{array} \end{array}$$

### Motion tracking

An LSTM RNN combined with a Kalman filter was developed for arm motion tracking (Figure 2). The measured shoulder coordinates are filtered using a Kalman filter, assuming no motion of the shoulder. The RNN input layer includes the measurements of the elbow and wrist joint locations from the Kinect sensor and the shoulder location from the Kalman filter. Two RNN versions were constructed, where the input to one of the networks additionally includes the uncalibrated elbow angle measured by the goniometer. The output layer includes the elbow and wrist coordinates. The LSTM has a two-step memory for all three joint locations (shoulder, elbow, wrist). For comparison, an additional run-time motion tracker based solely on a Kalman filter was programmed. The model used for the Kalman filter was based on assuming no shoulder motion, fixed lengths of the arm links (a simple rigid body arm model), and motion with a constant velocity of the elbow and wrist.

## Experiments

### Subjects and environments

The arm motion of eight healthy participants, mean age of 27.5 years (SD 1.77), six men and two women, was recorded (33). Participants had normal or corrected to normal vision. The Ben-Gurion University Human Subjects Research Committee approved the study, and informed consent was obtained from all subjects.

**Figure 3:**
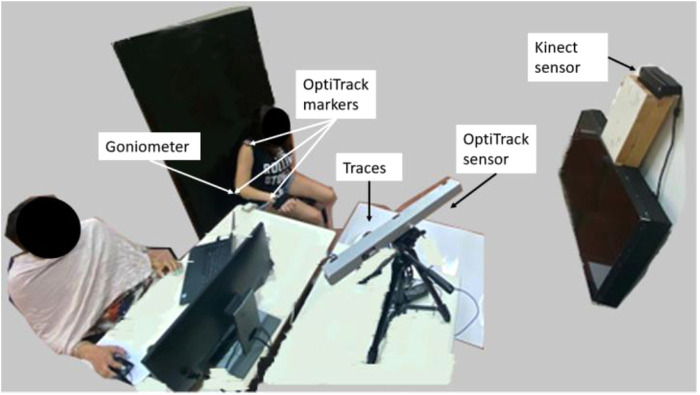
Experiment environment. The participant is seated on an armless chair with her hand strapped to the manipulator, about 2 m in front of the Kinect sensor. The goniometer and three V120:Trio reflectors are connected to the participant's arm.

The participants sat in a chair with a high backrest and no armrests, and their arm was strapped to a passive manipulator with three joints for supporting motion against gravity. The manipulator is modeled after an ergonomic computer desk armrest, and it immobilizes the wrist joint and elbow supination–pronation motion. The participant was free to perform elbow extension and shoulder motions. A Kinect sensor was placed in front of the participant (about 2 m away and about 0.5 m above the participant's shoulder). To demonstrate the ability to use multiple camera positions in the same training dataset, the Kinect was moved to an alternative location about 20 cm to the right in the same forward-facing orientation for one of the participants. An electronic twin axis goniometer (Biopac Systems, USA) was attached to the participant's arm above the elbow joint using double-sided tape (Figure 3). To facilitate fusion of the data from the two sensors (the Kinect and goniometer), the measurements from the sensors were acquired at synchronized time points. For testing the accuracy of the developed methods, the participant's motion was also tracked with a passive-marker motion capture system, V120:Trio (OptiTrack, USA). Three reflective markers were used for the V120:Trio recording. The markers were attached to bony landmarks on the right arm, i.e., acromion, lateral epicondyle, and wrist styloid process.

The recording environment was developed using Touch Designer (Derivative, CA). The environment was connected using a socket to MATLAB (Ver R2018b, MathWorks, USA), which was connected to the shared memory module of the electronic goniometer. The optimization procedure and the LSTM networks were programmed with Python (Ver 3.7) using the PyCharm interface (Ver 2019.2, JebBrains, USA). The statistical analysis was conducted with R using the RStudio interface (Ver 4.0.3, RStudio, Open Source). The data recording was conducted using an Intel i7-9700K 3.60 GHz processor and 32 GB RAM with a Windows 10 (64-bit) operating system. The training and analysis of the neural network were conducted using an Intel i7-8650U 2.11 GHz processor and 16 GB RAM with a Windows 10 (64-bit) operating system.

### Procedures

The data from the Kinect sensor and the goniometer were simultaneously recorded by the recording environment. The data from the V120:Trio sensor was recorded in parallel to another computer. At the beginning and end of each track, an additional (fourth) reflective marker was briefly exposed to the V120:Trio sensor by the experimenter to facilitate offline signal synchronization between the V120:Trio and the Kinect and goniometer. The sampling rates were 40 Hz for the Kinect, 100 Hz for the goniometer, and 120 Hz for the V120:Trio.

**Figure 4:**
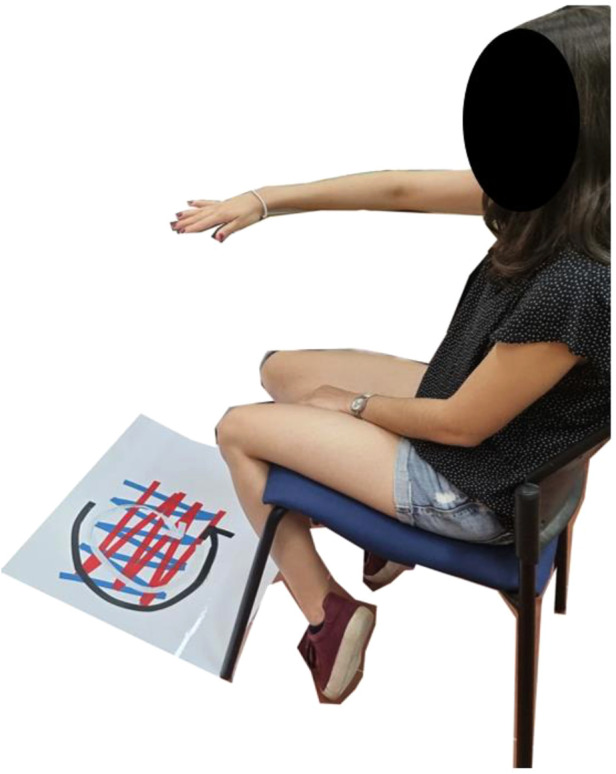
Color-coded tracks, blue: forward and backward, red: right and left, white: clockwise, black, counterclockwise.

The specific locations at which the goniometer is attached to the participant's arm can influence the relationship between the value measured and the true value of the elbow angle. Therefore, the goniometer must be calibrated after it is attached to the arm. Data for calibration were recorded at the beginning of each session. The participant was asked to keep his/her arm still in five different poses for 15 s each for the calibration recording. Following the calibration recording, the participant was asked to move his/her arm for eight 2-min intervals in one of four tracks marked by color-coded tape on the floor (Figure 4). The blue track indicated forward and backward movements, the red track indicated movements to the right and left, the white track indicated a clockwise circular movement, and the black track indicated a counterclockwise circular movement. Each track was repeated twice.

### Analyses

The recorded data underwent preprocessing. Calibration can be established with an absolute angle measurement device, e.g., an absolute goniometer. In the current experiment, the goniometer reading was calibrated to the V120:Trio reading to simplify testing system accuracy; however, this is not required in regular operation. The calibration was performed by calculating the average values of the V120:Trio and the goniometer at each of the five calibration poses. A linear regression model was fitted to these five data points. The regression model was used over all the recorded data to convert the goniometer readings to angles. Since the Kinect has the lowest sampling rate, the data from the goniometer and the V120:Trio were resampled based on the Kinect sampling time. The recordings of the goniometer and the Kinect were automatically synchronized through the recording environment. The V120:Trio signal was synchronized to the goniometer and Kinect data based on the recording of the fourth marker and manual scrutiny of the elbow angles (the angles calculated based on the V120:Trio data and the calibrated goniometer angles).

The optimization model fusing the Kinect and the goniometer measurements was solved using a constrained minimization of multivariate scalar functions programmed with the scipy.optimize^1^ package. The performance of the model was compared to the ground truth measurement. The LSTM network was programmed using the sequential neural network model in the tensorflow.keras^2^ package. The network was trained using the values established by the offline optimization algorithm. Its outcome was evaluated with respect to the recorded V120:Trio data. The data were arranged by tracks, and a four-fold cross-validation method was used. In each fold, a network was trained using the optimization data from six participants (48 tracks randomly presented), and the error was evaluated with respect to the V120:Trio data using the data from the remaining two participants (8 tracks each). Leaving subjects out for each fold was chosen to establish that the accuracy is not subject-dependent.

The network structure was based on an LSTM with 1 hidden layer and 50 neurons. The depth of the recurrent data memory was two. These values were empirically selected. The training method was based on the mean absolute error loss function and the efficient Adam stochastic gradient descent method. The model was fitted with 50 training epochs with a batch size of 72, so that the network was trained with small batches multiple times with a relatively short training time (below 0.5 h). The network tracking performance was compared both to the direct Kinect measurement and the tracking performance of a Kalman filter.

For performance analysis purposes, direct parameter measurements were regarded as the ground truth. Therefore, the marker locations obtained using V120:Trio were regarded as the ground truth for the joint locations, and the calibrated goniometer data were regarded as the ground truth for the elbow angle. As noted above, the two signals (the V120:Trio and goniometer) were synchronized at the beginning of each recording session to maintain coherency between the signals.

For the joint locations, the Euclidean distance to the ground truth was calculated for each sample. A constant error was expected between the position determined based on the Kinect measurement and the V120:Trio. Accordingly, for the joint positions, absolute residual errors were calculated. To this end, the average error was calculated and the absolute residual errors were determined for each subject by subtracting the average error from the Euclidean errors of each sample.

The goniometer elbow angle measurement was calibrated to the V120:Trio. Accordingly, both the optimization and the tracking networks were expected to produce the same angle as that measured by the V120:Trio. Therefore, absolute errors were calculated for the elbow angles. The angle calculated based on the Kalman filter tracking was not calibrated to the goniometer measurement, and, therefore, for the Kalman filter tracker, a constant difference in angles is expected and the absolute residual error was calculated.

## Results

### Optimization

The average absolute residual errors of the Kinect measurement were 35.0 mm (SD 28.7 mm) for the shoulder joint, 15.7 mm (SD 4.6 mm) for the elbow joint, and 75.6 mm (SD 27.3 mm) for the wrist joint. The average absolute residual errors of the offline optimization were 27.4 mm (SD 28.3 mm) for the shoulder joint, 15.5 mm (SD 8.8 mm) for the elbow joint, and 34.6 mm (SD 16.4 mm) for the wrist joint. The shoulder joint and the wrist joint position errors of the Kinect measurement were much larger than the offline optimization errors (shoulder: *F*_1,116,0.95 _=  5.9, *p* < 0.05, wrist: *F*_1,116,0.95_=  158.2, *p* < 0.001), while the elbow joint position errors were similar (Figure 4).

**Figure 5:**
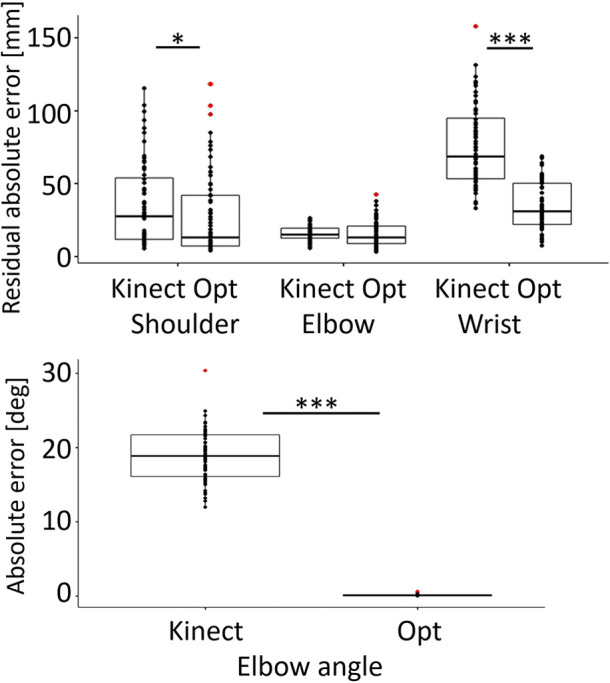
Top: Boxplot and data points for the absolute residual error with respect to the OptiTrack data of the shoulder, elbow, and wrist joint positions of the Kinect recording and the offline optimization. Bottom: Boxplot and data points of the absolute error of the elbow angle calculated from the Kinect measurements and from the offline optimization with respect to the goniometer measurements. Kinect: Kinect recording error, Opt: optimization error. Red points represent outliers. *p < 0.05, ***p < 0.001.

The average absolute error of the elbow angle calculated directly from the Kinect measurement was 18.9° (SD 3.4°). The error was much larger (*F*_1,116,0.95 _=  2559, *p* < 0.001) than the average absolute error of the elbow angle calculated based on the offline optimization, which was 0.1° (SD 0.1°) (Figure 5).

### Tracking

The position of the shoulder joint was determined using a Kalman filter in all tracking methods. Since the shoulder joint location is identically determined in the offline optimization scheme, the shoulder joint was not reanalyzed. The tracking errors for the subject recorded with the different Kinect positions were in the same range as those for the other subjects, in all tracking methods, for all joint positions, and for the elbow angle.

The average network training error (comparing the network output to the training data) for the network with the goniometer was 8.5 mm (SD 9.1 mm) for the elbow joint and 37.5 (SD 30.5 mm) for the wrist joint. The average network training error for the network without the goniometer was 8.6 mm (SD 9.5 mm) for the elbow joint and 31.3 (SD 25.5 mm) for the wrist joint.

**Figure 6:**
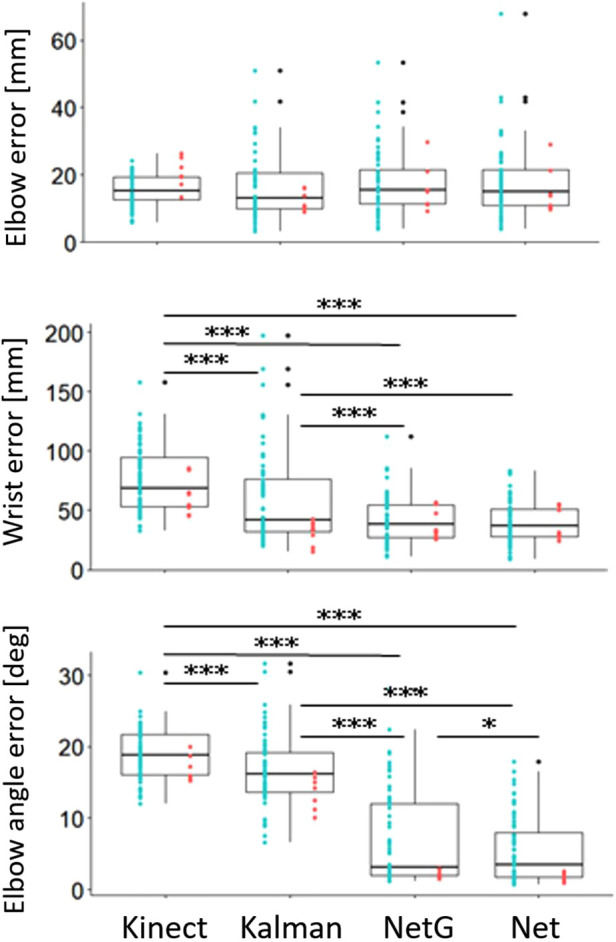
Boxplots and data points of the elbow joint absolute residual error (top), wrist joint absolute residual error (middle) and elbow angle absolute error (for the network trackers), and absolute residual error (for the Kalman filter) (bottom). Kinect: Kinect recording error, Kalman: Kalman filter error, NetG: network with goniometer input error, Net: network without goniometer input error. Black points represent outliers. Blue points represent data from the original position of the Kinect sensor. Red points represent data from the alternative position of the Kinect sensor. *p < 0.05, ***p < 0.001.

When compared to the ground truth recording (the V120:Trio or the calibrated goniometer), the average absolute residual errors for the elbow joint of the different trackers were similar to the errors of the direct Kinect recording. The errors were 16.3 mm (SD 9.6 mm) for the Kalman Filter, 17.6 mm (SD 9.5 mm) for the network with goniometer input, and 17.7 mm (SD 10.8 mm) for the network without the goniometer input (Figure 6).

The wrist joint position errors of the trackers were all lower than the error of the direct Kinect recording (Kalman filter: *F*_1,116,0.95 _=  21.95, *p* < 0.001, network with goniometer: *F*_1,116,0.95 _=  127, *p* < 0.001, network without goniometer: *F*_1,116,0.95 _=  139.5, *p* < 0.001). The Kalman filter had an average absolute residual error of 58.5 mm (SD 38 mm) and higher errors than both network trackers (network with goniometer: *F*_1,116,0.95 _=  17, *p* < 0.001, network without goniometer: *F*_1,116,0.95 _=  21.4, *p* < 0.001). The errors of the network trackers were similar, with an average of 42.5 mm (SD 20.6 mm) for the network with the goniometer and 40.1 mm (SD 27.3 mm) for the network without the goniometer.

The elbow angle errors of the run-time trackers were all lower than the error of the elbow angles calculated from the direct Kinect measurement (Kalman filter: *F*_1,116,0.95 _=  20.9, *p* < 0.001, network with goniometer: *F*_1,116,0.95 _=  346.1, *p* < 0.001, network without goniometer: *F*_1,116,0.95 _=  643.8, *p* < 0.001). The Kalman filter had an average absolute residual error of 16.6° (SD 5°), and its errors were higher than the absolute errors of both network trackers (network with goniometer: *F*_1,116,0.95 _=  207.5, *p* < 0.001, network without goniometer: *F*_1,116,0.95 _=  353.9, *p* < 0.001). The average absolute error for the tracker with the goniometer was 7.1° (SD 6.6°), which was higher (*F*_1,116, 0.95 _=  6.2, *p* < 0.05) than the average absolute error of 5.5° (SD 4.8°) for the tracker without the goniometer.

## Discussion

The offline optimization considerably improves the measurement accuracy. The error in the wrist position is reduced by more than 50%, and the elbow angle error is reduced by more than 10-fold to an average value of 0.1°. The low measurement errors following the optimization facilitate using the data as a training dataset for network trackers. The performance of the optimization method was validated for healthy subjects with a full range of arm motion. It will therefore also hold in case a subject has a limited range of motion.

Since the offline optimization uses a shoulder-centered kinematic model, data collected from multiple locations of the Kinect camera with respect to the participant can be analyzed collectively and can be used within a unified database for training a network for run-time tracking. The ability to use data from multiple locations facilitates simpler operation in multiple environments and in scenarios in which fixing a permanent location for the Kinect sensor may be difficult, e.g., due to the shared space between different systems. Such scenarios are typically encountered in rehabilitation clinics.

The biomechanical model used for the optimization was developed based on several system-specific constraints. Some of these constraints can be readily relaxed. For example, the model can be easily adapted for practicing with a manipulator that enables 3D motion of the hand or when trunk motion is not restricted. In many rehabilitation schemes for patients with stroke, as in the current work, the supporting manipulator constraints elbow supination–pronation motion. The Kinect skeletal model does not allow for direct measurement of forearm pronation/supination. In fact, with the Kinect skeleton model, elbow supination–pronation is manifested as the vertical displacement of the wrist (34). The current model may not suffice for training in which elbow supination–pronation is important, e.g., when grasping objects in different orientations. In such cases, alternative optimization methods, e.g., stochastic optimization, can be explored.

Measurements from an electronic goniometer are required when recording motion for the offline optimization algorithm. Goniometers are commonly available in rehabilitation clinics, and state-of-the-art electronic goniometers are lightweight and untethered (transmit measurements using Wi-Fi), therefore, the goniometer does not significantly impede motion. However, the goniometer must be attached to the participant's arm, which is less convenient than motion tracking with only a marker-less 3D video sensor. During run-time operation, the goniometer is not required, so to simplify operation, the goniometer can be used only during system setup for tuning the RNN to the new environment. In the current study, the goniometer was calibrated to the V120:Trio data to facilitate direct performance evaluation. This is not required in regular system operation, and in an actual deployment scenario, calibration can be established using any absolute angle measurement device, e.g., a standard manual goniometer. The calibration establishes a linear transformation between the goniometer's output and absolute elbow angle values. Establishing such a relation does not require spanning the full angle range or that the calibration angles measured are the same for all subjects. Therefore, adapting the calibration procedure for a subject with a reduced motion range is straightforward.

The network trackers trained with the data following the optimization had low tracking errors. The relatively short recurrent memory (2-steps) used, facilitates fast response to changes in motion velocity. The ability to closely follow the executed motion gives the network trackers an edge over the Kalman filter for rehabilitation training. Using a Kalman filter, forming a computationally fast, causal model that accurately represents human reaching motion is a challenge. The simplified model applied to the elbow and wrist motion for the Kalman filter in the current work does not capture motion onset and offset well, and this causes slow responses at the start and end of the reaching motion. Indeed, the LSTM network trackers outperformed the Kalman filter tracker in the tested trajectories. While the difference was small, the LSTM tracker without the goniometer had a lower elbow angle error than the LSTM tracker with the goniometer. The lower error of the LSTM without the goniometer input could be related to overfitting during network training. The low errors of the network tracker are promising, and the system may contribute to enhancing possible rehabilitation training schemes based on the Kinect sensor. The network performance determined is subject-independent (tested with a leave-subject-out procedure). However, the system was tested with healthy subjects and the tracker performance for individuals with stroke must be affirmed.

## Conclusion

The current study suggests an offline optimization method fusing measurements from a Kinect sensor and a goniometer based on an advanced biomechanical model. The optimization was used to generate training data for an LSTM network combined with a Kalman filter for run-time motion tracking. The methods considerably improved the accuracy of the joint location measurement for the shoulder and wrist joints and the accuracy of the calculated elbow angle. The Kinect measurements of the elbow joint location in the evaluated scenario were very accurate due to the characteristics of the movements, and the developed methods did not further improve (or degrade) it. The developed methods offer a low-cost, accurate motion tracking system that can easily be trained and adapted for rehabilitation clinics and home environments.

## Data Availability

The datasets presented in this study can be found in online repositories. The names of the repository/repositories and accession number(s) can be found below: Berman S, Agami S. Arm paths (Kinect, Goniometer, OptiTrack). *IEEE Dataport* (2022). doi: 10.21227/098c-vf55.

## Footnotes

^1^ https://docs.scipy.org/doc/scipy/reference/generated/scipy.optimize.minimize.html

^2^ https://www.tensorflow.org/api_docs/python/tf/keras/models
